# A rapid method for the preparation of an *in vitro* osteoporosis model of calf vertebrae: histological and biomechanical study

**DOI:** 10.3389/fbioe.2025.1527800

**Published:** 2025-02-05

**Authors:** Anli Shi, Yijie Liu, Qiang Ma, Jiaxin Li, Jiawang Fan, Zhaohui Ge

**Affiliations:** ^1^ The First Clinical Medical College of Ningxia Medical University, Yinchuan, China; ^2^ Department of Orthopaedics, General Hospital of Ningxia Medical University, Yinchuan, China; ^3^ College of Basic Medical Sciences, General Hospital of Ningxia Medical University, Yinchuan, China; ^4^ Department of Radiology, General Hospital of Ningxia Medical University, Yinchuan, China

**Keywords:** osteoporosis, lumbar spine, model, histology, biomechanics

## Abstract

**Background:**

*In vitro* biomechanical testing is crucial for the preclinical assessment of novel implant designs. Given the constraints of limited supply and high costs associated with human specimens, calf spines are frequently employed as surrogates for human spines in both *in vivo* and *in vitro* biomechanical studies.

**Methods:**

This study selected 60 spinal vertebrae from calves aged between 12 and 18 weeks. The specimens were randomly assigned to two treatment groups, A and B, each comprising 30 specimens. Group A served as the control without decalcification, while Group B underwent decalcification using an 18.3% ethylene diamine tetraacetic acid solution. The impact of decalcification was assessed through histological, imaging, and biomechanical analyses.

**Findings:**

Decalcification took approximately 2 months, resulting in osteoporotic vertebrae with a bone mineral density reduction of approximately 50.89% compared to pre-decalcification levels. The bone microstructure was significantly altered, characterized by a decrease in trabecular thickness and number and an increase in trabecular separation. Additionally, the trabecular bone pattern factor (TBPf) and Structure Model Index (SMI) increased. The modulus of elasticity, yield stress, and ultimate stress of the vertebral bodies were all reduced in correlation with the decrease in bone mineral density, demonstrating a strong correlation between these parameters.

**Interpretation:**

The data from this study indicate that the decalcification method is effective and capable of rapidly establishing an osteoporotic model suitable for biomechanical testing of clinical devices. This method offers the benefits of ease of operation, reliability, and a controllable degree of osteoporosis.

## Introduction

Studies have demonstrated that examining the internal fixation of osteoporotic (OP) vertebrae is crucial for comprehending bone mechanics and the advancement of innovative and superior implant designs (Wang and Guo, 2020). In mechanical experiments concerning spinal fusion and instrumentation techniques, the trial production of surgical instruments necessitates extensive testing on various animal models. Consequently, the accurate selection and preparation of an exemplary animal model for OP experiments, capable of providing numerous reproducible tests, are fundamental to conducting biomechanical studies on osteoporosis. Human spinal specimens and those from non-human primates, such as orangutans, are often considered. However, their high cost, limited reproducibility, and ethical concerns significantly restrict their application as experimental models (Wang and Guo, 2020; Wilke et al., 2023).

Swartz et al. (1991) established that the calf spine possesses tissue density, equivalent mineral density, apparent density, ash density and content, compressive modulus, and strength that are analogous to those of a young, healthy human spine. Moreover, the lumbar vertebrae are sufficiently large for the assessment of spinal implants (Riley et al., 2004). As a result, in the realm of biomechanical research, the calf spine is frequently employed as an alternative to human specimens for both *in vivo* and *in vitro* experiments, including spinal fusion and instrumentation techniques (Sait et al., 2016; Akgül et al., 2020; Thomas et al., 2011; Chaudhari et al., 2011).

The preparation of calf osteoporosis (OP) models encompasses various techniques such as gene knockout (Alonso-Perez et al., 2018), hormone induction (Chen et al., 2017), low-calcium diets, and oophorectomy (Rondanelli et al., 2021). However, these methods typically require over 6 months to develop, which, when combined with the procurement of live animals, their maintenance, sterilization, and pharmacological manipulations, leads to an extended modeling cycle and a complex process. The limited reliability of these models (Chen et al., 2017; Rondanelli et al., 2021; Eastell et al., 2019) falls short of the demands for repeated animal experiments, particularly for the biomechanical assessment of spinal instrumentation within a short timeframe using a large number of animal models. In light of this, this study focused on calf vertebrae and employed the decalcifying agent ethylene diamine tetraacetic acid (EDTA) to decrease the mineral content of bovine vertebrae (Sultan and Jayash, 2023). By doing so, we aimed to alter the bone mineral density (BMD) and biomechanical properties, thereby accurately mimicking the spine of an osteoporotic patient in a biomechanical context.

## Materials and methods

### Preparation of spinal specimens

Sixty lumbar vertebrae from calves aged between 12 and 18 weeks were carefully selected for this study. The research was approved by the Ethics Committee of the General Hospital of Ningxia Medical University (KYLL-2022-1137) and adhered strictly to the ARRIVE guidelines. It was conducted in accordance with the United Kingdom Animals Act 1986 and its associated guidelines, ensuring that all experimental animals received humane care in compliance with the Animal Welfare Act. During the radiographic evaluation, vertebrae with bone defects or deformities were excluded from the study. The surrounding muscle tissue of the vertebral body was meticulously removed. A portion of the transverse process, the spinous process, and the complete periosteum were preserved. The specimens were then randomly assigned to two treatment groups, A and B, each consisting of 30 specimens. Group A served as the control group, with no decalcification process applied, whereas Group B underwent decalcification using an EDTA solution for a duration of 8 weeks.

### Decalcification procedure

EDTA (Beijing Boao Technology Co., Ltd.), weighing 368 g, and NaOH (Xuzhou Tianhong Chemical Co., Ltd.), weighing 156 g, were combined with approximately 1800 mL of PBS solution to ensure complete dissolution. The resulting solution was then diluted to a final volume of 2000 mL. The concentration of the solution was adjusted to 18.3%, and the pH was calibrated to 7.4. Group B was submerged in this prepared 18.3% EDTA solution for decalcification. To maintain a consistent EDTA concentration, the soaking solution was refreshed daily. BMD was determined both before and after the immersion process using a dual-energy X-ray absorptiometry (DEXA) scanner (syngo Osteo CT; Siemens). Osteopenia is defined as a reduction in the average BMD of the lumbar spine to between 75% and 87% of the mean. Osteoporosis is characterized by a BMD that is less than 75% of the average, and severe osteoporosis is indicated when the BMD is below 63% of the average (Egermann et al., 2005). Once the regional BMD reached the desired levels, the vertebrae were preserved in the 10% formalin solution.

### Imaging and histological examinations

A variety of parameters, including trabecular thickness (Tb.Th), trabecular number (Tb.N), bone volume to total volume ratio (BV/TV), trabecular separation (Tb.Sp), bone surface to bone volume ratio (BS/BV), trabecular bone pattern factor (TBPf), and Structure Model Index (SMI), were assessed using micro-CT (SkyScan 1176; Belgium) in each group, both before and after decalcification. Meanwhile, changes are observed in microstructures such as trabecular bones in tissues before and after decalcification in groups A and B through HE staining.

### Biomechanical testing

**FIGURE 1:**
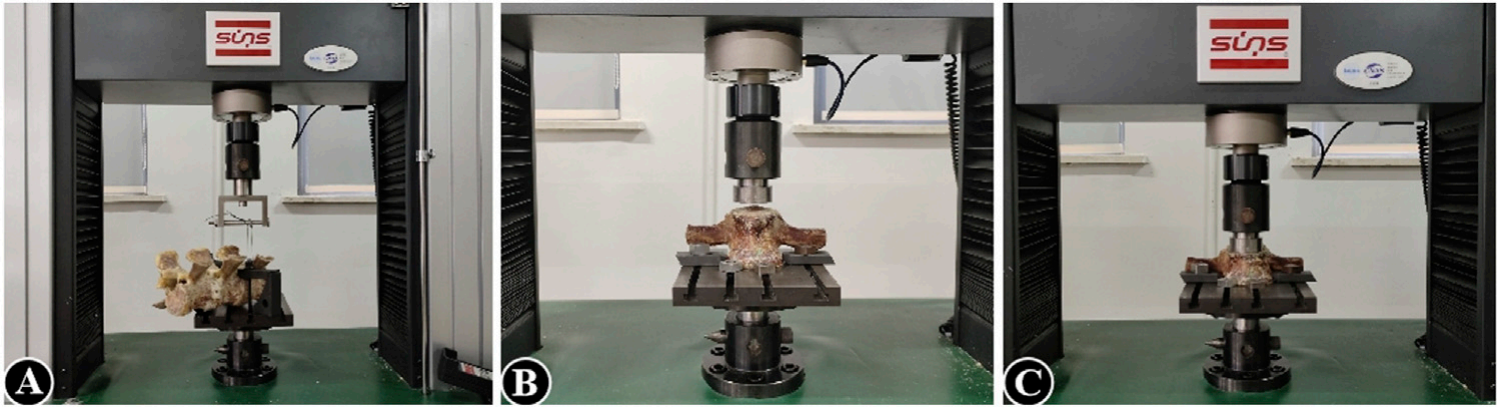
**(A)** Pedicle screw extraction experiment of vertebral specimens in the decalcification group; **(B, C)** axial compression experiment of vertebral specimens in the decalcification group.

The mean axial pull-out force (F_max_) of the pedicle screws in the lumbar vertebra for each specimen was measured using a tensile/torsion tester (DYNA-MESS, Germany), and the correlation between F_max_ and the degree of BMD was also investigated (Figure 1A). After fixing the bottom and connecting it to the same experimental equipment, axial load is applied to the top of the lumbar vertebra to test for elastic modulus, yield stress, stress decrease, and the ultimate stress and to observe their correlations with BMD, respectively (Figures 1B, C). Adhering to the established protocol, the samples were kept in a humid state by consistently misting them with a 0.9% saline solution throughout the duration of the testing. Furthermore, all experimental procedures were conducted at a precisely controlled ambient temperature of 25°C (Wang et al., 2024).

### Statistical analysis

In this study, statistical analysis was conducted using SPSS software (version 24.0, SPSS Inc., Chicago, IL, United States). The measurement data were expressed as the mean ± standard deviation (SD). After the normality test, repeated measures analysis of variance was employed to assess the differences over time within the same group. Additionally, Pearson correlation analysis was utilized to evaluate the relationship between BMD and key mechanical properties, including the maximum pull-out force (F_max_), elastic modulus, yield stress, and ultimate stress. A p-value of less than 0.05 was set as the threshold for determining statistical significance.

## Results

### Changes in vertebral BMD

**FIGURE 2:**
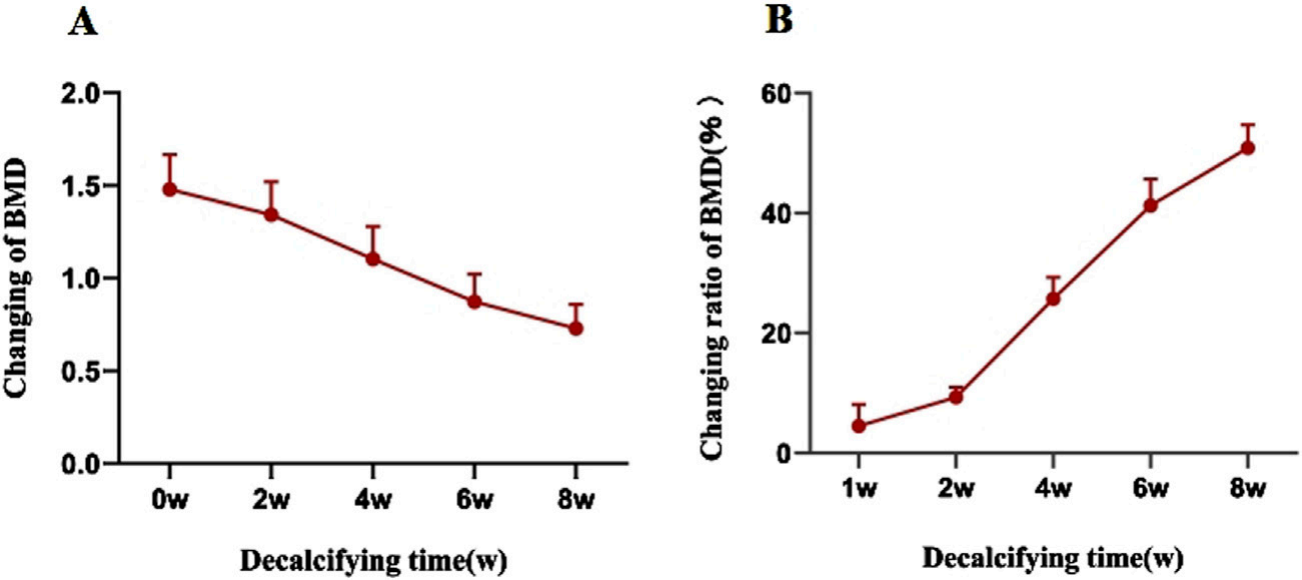
**(A)** The BMD of vertebral specimens in the decalcification group gradually decreased with an increase in the decalcification time. **(B)** Rate of change in the BMD of vertebral specimens in the decalcification group in relation to the decalcification time.

The average BMD of Group B prior to decalcification was recorded at (1.48 ± 0.19) g/cm^2^. Following an 8-week decalcification period, all vertebral bodies in Group B were found to have reached osteoporotic levels, with the average BMD decreasing by 50.89% relative to the baseline values (prior to decalcification), which was a statistically significant reduction (P < 0.001). The repeated measures analysis of variance confirmed that the difference in BMD of the vertebral specimens before and after decalcification within Group B was highly statistically significant (F = 740.743 and P < 0.001) (Table 1; Figure 2).

**TABLE 1:** Microarchitectural parameters obtained in non-decalcified and decalcified vertebrae.

Parameter	Non-decalcified	1-Mon decalcified	2-Mon decalcified	2-Mon decalcified vs. non-decalcified (%)	*P-*value
BMD (g/cm^2^)	1.48 ± 0.19	1.12 ± 0.20	0.73 ± 0.13	−50.89	<0.001
Tb.Th (mm)	0.40 ± 0.04	0.28 ± 0.031	0.24 ± 0.03	−40.96	<0.001
Tb.N (mm^−1^)	1.79 ± 0.09	1.07 ± 0.04	0.73 ± 0.14	−59.47	<0.001
Tb.Sp (mm)	0.66 ± 0.11	1.38 ± 0.26	2.13 ± 0.28	64.72	<0.001
BS/BV (mm^2^)	11.99 ± 0.68	19.79 ± 1.03	25.16 ± 1.62	109.94	<0.001
BV/TV (%)	16.39 ± 0.78	8.89 ± 0.72	5.87 ± 0.65	−64.19	<0.001
TBPf	−2.10 ± 0.66	2.60 ± 0.33	3.86 ± 0.52	284.14	<0.001
SMI	0.65 ± 0.26	2.50 ± 0.76	3.65 ± 0.67	461.27	<0.001

### Image and histomorphometric changes

**FIGURE 3:**
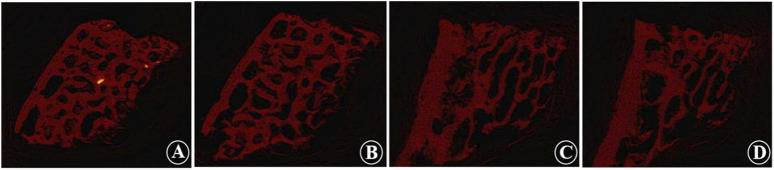
Micro-CT images show the trabecular morphology of the calf lumbar vertebrae before and after decalcification. **(A, B)** Non-decalcified vertebrae, which show that the trabecular structure is dense, uniformly distributed, and interconnected to form a meshwork. **(C, D)** Decalcified vertebrae, which show that the bone structure is formed loosely, and some of the trabecular joints appear broken, with the distance between the trabecular joints widened and the trabecular components of the bone thinned out and fewer in number.

**FIGURE 4:**
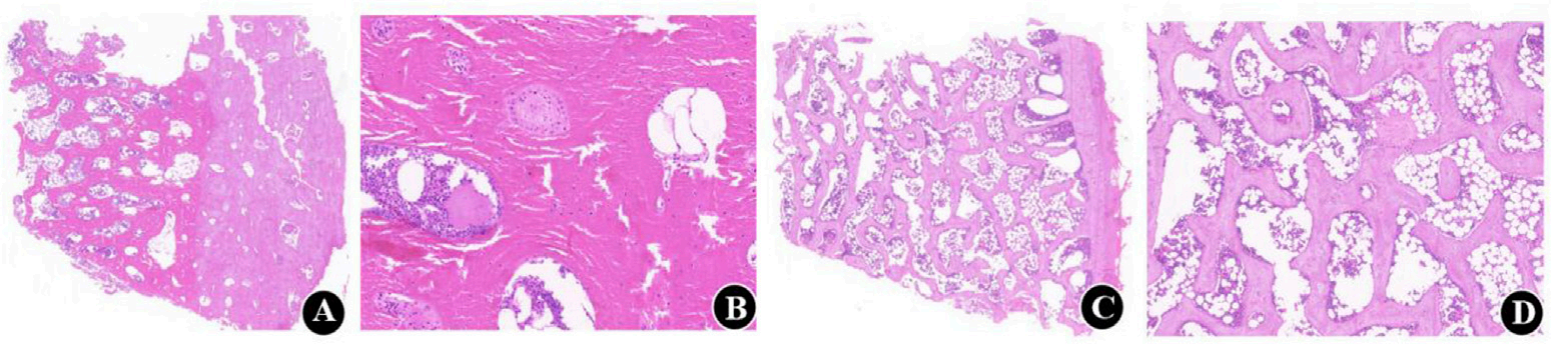
Five-micrometer-thick undecalcified and decalcified sections of calf vertebrae. **(A, B)** Histological sections before decalcification. H and E, original magnifications are × 1x (scale bar, 1000 µm) and × 5x (scale bar, 200 µm), respectively; (**C, D)** Histological sections at 6 weeks of decalcification. H and E, original magnifications are × 1x (scale bar. 1000 µm) and × 5x (scale bar, 200 µm), respectively.

Micro-CT analysis revealed significant changes in the trabecular bone microarchitecture of Group B specimens after an 8-week decalcification period. Specifically, there were substantial decreases in Tb.Th by −40.96%, Tb.N by −59.47%, and BV/TV by −64.19%. Concurrently, there were significant increases in Tb.Sp by 64.72%, BS/BV separation rate by 109.94%, TBPf by 284.14%, and SMI by 461.27%. All these changes were statistically significant (P < 0.001) (Table 1; Figure 3). The histological sections of Group B demonstrated a progressive thinning of the trabecular bone, a reduction in the number of trabeculae, fractures within the trabecular bone, and an enlargement of the bone marrow cavity (Figure 4).

### Axial tension test results

**FIGURE 5:**
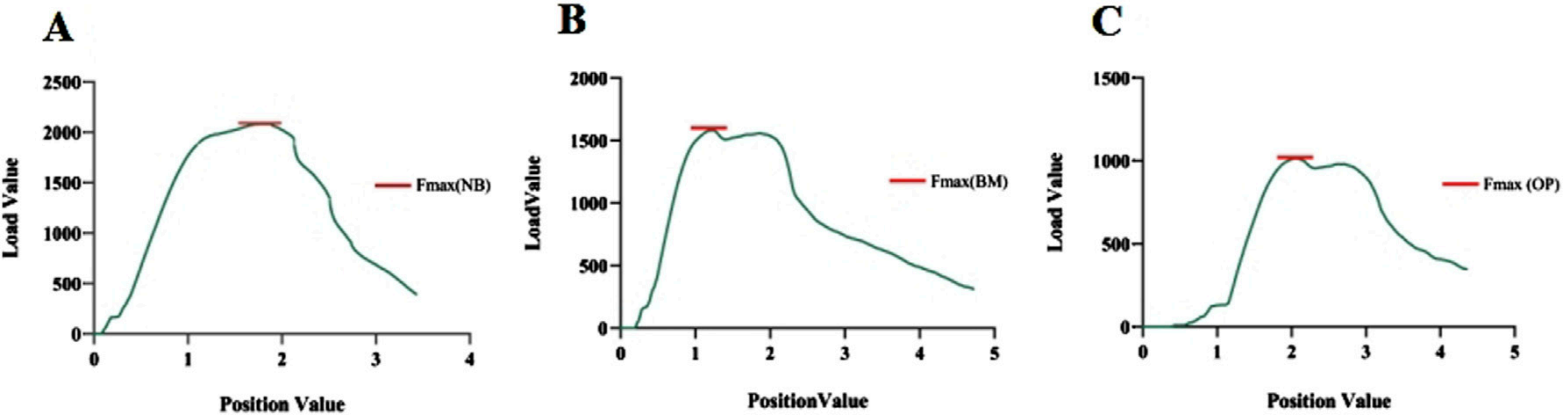
**(A–C)** Maximum pedicle screw extraction forces under normal bone (NB) quality, reduced bone mass (BM), and osteoporotic (OP) conditions, respectively.

**FIGURE 6:**
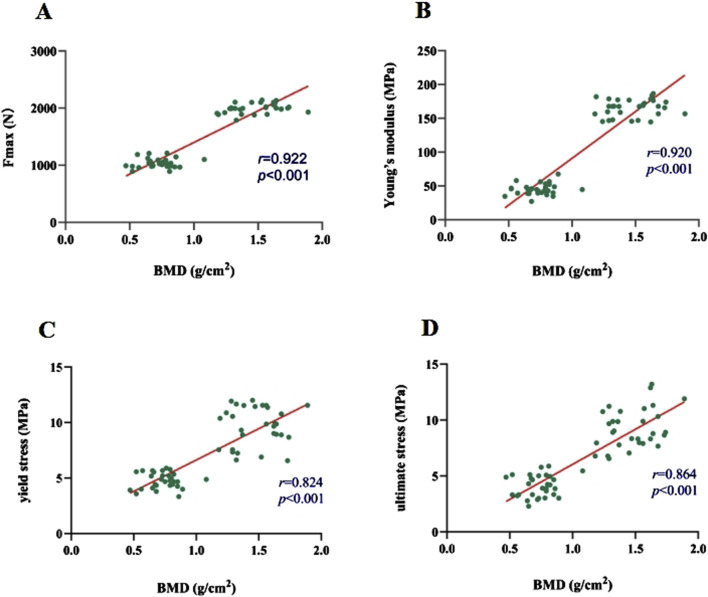
**(A–D)** Correlations of F_max_, modulus of elasticity, yield stress, and ultimate stress with BMD, respectively.

The average maximum axial pull-out force (F_max_) values for groups A and B was 1989.40 ± 116.90 N and 1995.68 ± 83.85 N, respectively. There was no statistically significant difference between the two groups (t = 0.250 and P = 0.803). However, within Group B, F_max_ decreased as the duration of decalcification increased, which was a statistically significant trend (F = 1704.65 and P < 0.001) (Table 2; Figure 5). Correlation analysis between BMD and F_max_ across the two groups revealed a positive correlation (r = 0.922 and P < 0.001), indicating that as BMD increased, F_max_ also increased (Figure 6A).

**TABLE 2:** Biomechanical properties obtained in non-decalcified and decalcified vertebrae.

Parameter	Non-decalcified	1-Mon decalcified	2-Mon decalcified	2-Mon decalcified vs. non-decalcified (%)	*P*-value
Young’s modulus (MPa)	165.48 ± 12.51	93.96 ± 12.05	45.08 ± 8.08	−72.83	0.002
Yield stress (MPa)	9.67 ± 1.75	6.82 ± 1.15	4.76 ± 0.71	−49.24	<0.001
Ultimate stress (MPa)	9.28 ± 1.78	6.46 ± 1.43	4.10 ± 0.98	−55.72	<0.001
F_max_ (N)	1995.68 ± 83.85	1,322.90 ± 80.64	1,302.87 ± 82.19	−48.27	<0.001

### Vertebral compression test results

**FIGURE 7:**
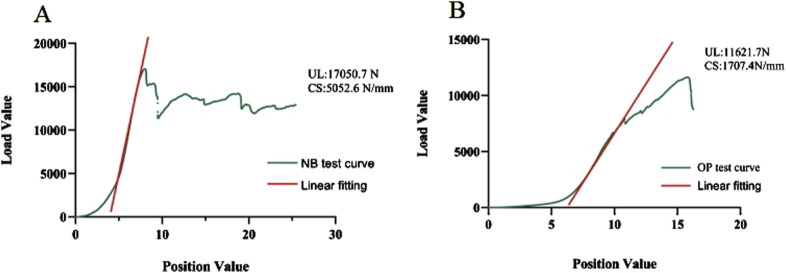
**(A, B)** UL and CS under normal bone quality and osteoporotic conditions, respectively.

The axial compression test results for the vertebral bodies in Group B indicated significant reductions in mechanical properties compared to Group A. Specifically, the elastic modulus decreased by 72.83%, the yield stress decreased by 49.24%, and the ultimate stress decreased by 55.72% (Table 2). For Group A, under normal bone quality conditions, the ultimate load (UL) and compression stiffness (CS) were 17,050.7 N and 5,052.6 N/mm, respectively. These values were significantly higher than those of the osteoporotic group, which were 11,021.7 N and 1707.4 N/mm, respectively (Figure 7). As anticipated, the elastic modulus (r = 0.920 and P < 0.001), yield stress (r = 0.824 and P < 0.001), and ultimate stress (r = 0.864 and P < 0.001) of the calf vertebrae demonstrated strong positive correlations with vertebral BMD (Figures 6B–D). This suggests that higher BMD is associated with greater mechanical strength and stiffness in the vertebrae.

## Discussion

Biomechanical *in vitro* testing plays a crucial role in the preclinical evaluation of new implants (Wang and Guo, 2020). Animal models are frequently employed as an alternative or supplement to human specimens in these tests, aiming to reduce specimen costs and variability (Wilke et al., 2023). Among various animal models, the calf lumbar spine is often selected for studying the stabilizing effects of spinal instrumentation systems due to its similarity to human spine characteristics in both quasi-static and cyclic tests (Sunni et al., 2020; Cotterill et al., 1986). Thus, in this study, the calf lumbar spine was utilized as a surrogate for the human spine in biomechanical research.

In the present study, an 18.3% mmol/L EDTA solution was employed to remove calcium and certain minerals within the vertebral body, thereby facilitating decalcification and a reduction in bone biomechanical properties. The neutral pH of the EDTA solution, coupled with its slower decalcification rate compared to acidic solutions like hydrochloric acid, offers greater stability and accuracy in binding with calcium, causing minimal tissue damage and preserving tissue cell structure integrity (Freiet al., 2022). Decalcification progress in this study showed a gradual decrease in BMD, with a slower rate initially, a more rapid change between weeks 2 and 4, and a subsequent slowdown. This may be related to the threshold of the EDTA decalcification process, that is, when a large number of minerals in the bone are chelated and free, decalcification is accelerated, and in the later stage, the change in BMD tends to be slow due to the small amount of mineral remaining. BMD of the decalcification group decreased by approximately 12.56% in the first week and 21.68% in the fourth week post-intervention. By the sixth week, BMD was 28.5% lower than pre-decalcification levels, meeting the diagnostic criteria for OP. After 8 weeks, all vertebrae in Group B reached osteoporotic levels, with an average BMD decrease of 48.9% compared to pre-decalcification values. This indicates that varying degrees of OP models can be replicated by adjusting the duration of EDTA immersion, reducing BMD by approximately 10%–50% over 1–8 weeks.

In the context of bone microstructure, the structural parameters of the trabecular bone are intimately linked to mechanical properties (Amirtham et al., 2020). Bone loss alone does not fully account for the decreased bone strength and increased brittleness observed in OP patients. Studies have revealed significant changes in trabecular structure, even with minor alterations in the trabecular volume fraction (Guenoun et al., 2017). Notably, the main trabeculae in calf lumbar spines are concentrated along the longitudinal axis, similar to humans, and are crucial for weight-bearing. Studies on vertebral trabeculae in OP patients have found that while horizontal trabeculae are shorter and thinner than vertical trabeculae, they contribute more significantly to bone structure strength (Amirtham et al., 2020). As bone mass decreases, horizontal trabeculae are preferentially lost, leading to excessive loads on the remaining vertical trabeculae and subsequent compensatory hypertrophy (Lee et al., 2011). Micro-CT analysis after 2 months of decalcification showed significantly lower values for Tb.Th, Tb.N, and BV/TV in the decalcified group compared to Group A, with higher increases in BS/BV, Tb.Sp, TBPf, and SMI. These results indicate a significant BMD reduction in Group B, with trabecular structural parameter changes possibly related to trabecular perforation and the discontinuity of the trabecular meshwork. The higher TBPf value in Group B after 2 months confirms EDTA’s role in reducing trabecular bone interconnection (Cesar et al., 2020). The loss of trabecular connectivity leads to a disproportionate loss of cancellous bone relative to cortical bone, an area of importance in OP fracture risk studies (Ozan et al., 2017). Based on the abovementioned results, we can infer that the loss of bone mass elements caused by trabecular perforation is the main mechanism of structural changes in the OP model. The decrease in bone strength caused by changes in the trabecular structure may contribute more to bone loss (Lee et al., 2011).

Studies have demonstrated that the number of trabecular bones directly affects cortical bone mechanical strength and the screw fixation ability (Wang and Guo, 2020). The statistical results in this study showed a decrease in F_max_ for Group B’s vertebrae with prolonged decalcification time. The correlation analysis between BMD and F_max_ across the two groups revealed a significant positive correlation. The axial compression test for Group B’s vertebral bodies showed a 72.83% decrease in elastic modulus, a 49.24% decrease in yield stress, and a 55.72% decrease in ultimate stress compared to Group A. As expected, Young’s modulus, yield stress, and ultimate stress of calf vertebrae were strongly positively correlated with vertebral BMD, further confirming that BMD reduction directly affects the fixation strength of pedicle screws and the compressive strength of vertebral bodies. In conclusion, while bone mass is lost, the mechanical properties of trabecular bone also change, playing a significant role in increased bone fragility and fracture risk in osteoporotic patients. Studying the microstructure and composition of trabecular bone aids in accurately evaluating its mechanical properties, which is crucial for preventing osteoporotic fractures.

Several limitations to this study should be acknowledged. First, the type, size, drying degree, and environmental medium of the samples can influence experimental outcomes. Second, there is an inevitable end-plate error between the sample and the measuring instrument due to interface friction. Third, the absence of intermediate measurements during the 8-week decalcification process means that we cannot dynamically track the changes in BMD and microarchitecture. Lastly, the sample size used in this experiment is small, and further research with a larger sample size is necessary to ensure the accuracy and effectiveness of the findings.

## Conclusion

The process of immersing the calf lumbar spine in EDTA to replicate *in vivo* decalcification is an effective approach for rapidly establishing an osteoporosis model suitable for spinal biomechanics research. This method offers several benefits, such as a short modeling cycle, high repeatability, and the capacity to control the level of osteoporosis, making it an advantageous technique for these types of studies.

## Data Availability

The raw data supporting the conclusions of this article will be made available by the authors, without undue reservation.

## References

[B1] Akgül T. Korkmaz M. Pehlivanoglu T. Bayram S. Özdemir M. A. Karalar Ş. (2020). Biomechanical comparison of pull-out strength of different cementation and pedicle screw placement techniques in a calf spine model. Indian J. Orthop. 54 (Suppl. 1), 134–140. 10.1007/s43465-020-00199-z 32952921 PMC7474045

[B2] Alonso-Perez A. Franco-Trepat E. Guillan-Fresco M. Jorge-Mora A. Lopez V. Pino J. (2018). Role of toll-like receptor 4 on osteoblast metabolism and function. Front. Physiol. 9, 504. 10.3389/fphys.2018.00504 29867550 PMC5952219

[B3] Amirtham S. M. Ozbey O. Kachroo U. Ramasamy B. Vinod E. (2020). Optimization of immunohistochemical detection of collagen type II in osteochondral sections by comparing decalcification and antigen retrieval agent combinations. Clin. Anat. 33, 343–349. 10.1002/ca.23441 31381185

[B4] Cesar R. Bravo-Castillero J. Ramos R. R. Pereira C. Zanin H. Rollo J. (2020). Relating mechanical properties of vertebral trabecular bones to osteoporosis. Comput. Methods Biomech. Biomed. Engin 23, 54–68. 10.1080/10255842.2019.1699542 31813291

[B5] Chaudhari R. Zheng X. Wu C. Mehbod A. A. Transfeldt E. E. Winter R. B. (2011). Effect of number of fusion levels on S1 screws in long fusion construct in a calf spine model. Spine (Phila Pa 1976) 36 (8), 624–629. 10.1097/BRS.0b013e3181d99d9b 21178830

[B6] Chen J. Liu W. Zhao J. Sun C. Chen J. Hu K. (2017). Gelatin microspheres containing calcitonin gene-related peptide or substance P repair bone defects in osteoporotic rabbits. Biotechnol. Lett. 39, 465–472. 10.1007/s10529-016-2263-4 27909823

[B7] Cotterill P. C. Kostuik J. P. D'Angelo G. Fernie G. R. Maki B. E. (1986). An anatomical comparison of the human and bovine thoracolumbar spine. J. Orthop. Res. 4, 298–303. 10.1002/jor.1100040306 3734937

[B8] Eastell R. Rosen C. J. Black D. M. Cheung A. M. Murad M. H. Shoback D. (2019). Pharmacological management of osteoporosis in postmenopausal women: an endocrine society* clinical practice guideline. J. Clin. Endocrinol. Metab. 104, 1595–1622. 10.1210/jc.2019-00221 30907953

[B9] Egermann M. Goldhahn J. Schneider E. (2005). Animal models for fracture treatment in osteoporosis. Osteoporos. Int. 16 (Suppl. 2), S129–S138. 10.1007/s00198-005-1859-7 15750681

[B10] Freitas E. C. Dalmolin S. P. Da S. M. de Oliveira F. H. Pilar E. (2022). Evaluation of EDTA and nitric acid solutions for decalcification of joints in AG/WT, BALB/c, C57, DBA1/J mice, and in Wistar rats. Biotech. Histochem 97, 372–381. 10.1080/10520295.2021.2003431 34845957

[B11] Guenoun D. Foure A. Pithioux M. Guis S. Le Corroller T. Mattei J. P. (2017). Correlative analysis of vertebral trabecular bone microarchitecture and mechanical properties: a combined ultra-high field (7 tesla) mri and biomechanical investigation. Spine (Phila Pa 1976) 42, E1165–E1172. 10.1097/BRS.0000000000002163 28338579

[B12] Lee C. Y. Chan S. H. Lai H. Y. Lee S. T. (2011). A method to develop an *in vitro* osteoporosis model of porcine vertebrae: histological and biomechanical study. J. Neurosurg. Spine 14, 789–798. 10.3171/2010.12.SPINE10453 21395393

[B13] Ozan F. Pekedis M. Koyuncu S. Altay T. Yildiz H. Kayali C. (2017). Micro-computed tomography and mechanical evaluation of trabecular bone structure in osteopenic and osteoporotic fractures. J. Orthop. Surg. Hong. Kong 25, 2309499017692718. 10.1177/2309499017692718 28215116

[B14] Riley L. R. Eck J. C. Yoshida H. Koh Y. D. You J. W. Lim T. H. (2004). A biomechanical comparison of calf versus cadaver lumbar spine models. Spine (Phila Pa 1976) 29, E217–E220. 10.1097/00007632-200406010-00021 15167671

[B15] Rondanelli M. Perdoni F. Peroni G. Caporali R. Gasparri C. Riva A. (2021). Ideal food pyramid for patients with rheumatoid arthritis: a narrative review. Clin. Nutr. 40, 661–689. 10.1016/j.clnu.2020.08.020 32928578

[B16] Sait A. Prabhav N. R. Sekharappa V. Rajan R. Raj N. A. David K. S. (2016). Biomechanical comparison of short-segment posterior fixation including the fractured level and circumferential fixation for unstable burst fractures of the lumbar spine in a calf spine model. J. Neurosurg. Spine 25 (5), 602–609. 10.3171/2016.4.spine1671 27285665

[B17] Sultan N. Jayash S. N. (2023). Evaluation of osteogenic potential of demineralized dentin matrix hydrogel for bone formation. BMC Oral Health 23 (1), 247. 10.1186/s12903-023-02928-w 37118728 PMC10148431

[B18] Sunni N. Askin G. N. Labrom R. D. Izatt M. T. Pearcy M. J. Adam C. J. (2020). The effect of vertebral body stapling on spine biomechanics and structure using a bovine model. Clin. Biomech. (Bristol, Avon) 74, 73–78. 10.1016/j.clinbiomech.2020.02.006 32145672

[B19] Swartz D. E. Wittenberg R. H. Shea M. White A. R. Hayes W. C. (1991). Physical and mechanical properties of calf lumbosacral trabecular bone. J. Biomech. 24, 1059–1068. 10.1016/0021-9290(91)90022-f 1761582

[B20] Thomas A. Kepler C. K. Meyers K. Green D. W. Wright T. M. Rawlins B. A. (2011). The effect of sacral decortication on lumbosacral fixation in a calf spine model. Spine (Phila Pa 1976) 36 (6), E388–E392. 10.1097/BRS.0b013e3181f54f23 21270709

[B21] Wang Q. D. Guo L. X. (2020). Biomechanical role of osteoporosis in the vibration characteristics of human spine after lumbar interbody fusion. Int. J. Numer. Method Biomed. Eng. 36, e3402. 10.1002/cnm.3402 33021071

[B22] Wang Z. Yang W. Liu X. Liang S. Cai Z. Guo W. (2024). An *in vitro* biomechanical evaluation of integrated lateral plate combined with oblique lateral interbody fusion in different bone conditions. Sci. Rep. 14 (1), 29432. 10.1038/s41598-024-80631-8 39604491 PMC11603067

[B23] Wilke H. J. Betz V. M. Kienle A. (2023). Biomechanical *in vitro* evaluation of the kangaroo spine in comparison with human spinal data. J. Anat. 243, 128–137. 10.1111/joa.13852 36929138 PMC10273331

